# Echocardiographic assessment of left atrial size in patients with end-stage renal disease

**Published:** 2009-05

**Authors:** Dardan Koçinaj, Masar Gashi, Allma Koçinaj, Hajrije Korça, Merita Berisha, Naser Ramadani

**Affiliations:** University Clinical Centre of Kosova, Prishtina, Kosova; University Clinical Centre of Kosova, Prishtina, Kosova; University Clinical Centre of Kosova, Prishtina, Kosova; University Clinical Centre of Kosova, Prishtina, Kosova; National Institute of Public Health of Kosova, Prishtina, Kosova; National Institute of Public Health of Kosova, Prishtina, Kosova

## Abstract

**Background:**

Cardiac disease is the most common cause of death in patients with end-stage renal disease. It is assumed that the high rate of cardiovascular mortality is related to accelerated atherosclerosis. Patients with chronic renal insufficiency have an increased prevalence of coronary artery disease, silent myocardial ischaemia, complex ventricular arrhythmias, atrial fibrillation, left ventricular hypertrophy, annular mitral and aortic valve calcification, and enlargement of the left atrium, than patients with normal renal function. It is also well known that haemodialysis is associated with cardiovascular structural changes and rapid fluctuations in electrolyte levels.

In this study, we sought to estimate left atrial size by means of echocardiography and to determine any correlations between different echocardiographic measurements in patients with end-stage renal disease.

**Methods:**

We analysed data from 123 patients who were on regular haemodialysis, by means of traditional transthoracic echocardiographic examination. The usual statistical parameters, correlations and the Student’s *t*-test were performed, with levels of significance of *p* < 0.01 and *p* < 0.05.

**Results:**

The most presented age group was 60 to 69 years old, with a predomination of females (56.1%). We found dilated left atrium in 26.02% of the study patients and a high statistical correlation between different methods of measurement and calculated volumes of the left atrium.

**Conclusion:**

Evaluation of left atrial size should be determined by several different measurements, and left atrial enlargement should be seen as a risk factor for advancing disease.

## Summary

Cardiovascular morbidity and mortality are high in patients with end-stage renal disease. These patients have an increased prevalence of coronary heart disease, silent myocardial ischaemia, ventricular and supraventricular disturbances in heart rhythm,^1^ left ventricular hypertrophy, changes in the mitral and aortic valves, and enlargement of the left atrium.^2^-^4^ Left atrial size has a prognostic importance in heart conditions. Although two-dimensional echocardiographic data are traditionally used to estimate left atrial size (diameter, area or volume),^5^ no specific echocardiographic technique is universally accepted for the determination of atrial size.^6^

The repercussions of high blood pressure are apparent in the heart as increased volume and hypertrophy of the left ventricle. The size of the left atrium is also a predictor of cerebrovascular insults and atrial fibrillation,^7^ but there are conflicting data on the effect of high arterial pressure alone on the size of the left atrium.^8^

Diastolic dysfunction of the left ventricle plays an important role in the pathogenesis of symptoms in patients with hypertrophic cardiomyopathy. Abnormal filling of the left ventricle^9^ and mitral regurgitation also result in increased left atrial size.^10^ Atrial fibrillation may be associated with increased activation of atrial natriuretic peptides, but atrial volume, wall pressure and strain are also important factors in natriuretic peptide activation.^11^

A significant correlation has been found between several methods of echocardiographic (ECG) measurement of the left atrium, but size of the body and thorax were not found to affect the data. Evaluation of atrial size should be done with ECG measurements obtained from different viewpoints.^12^ Magnetic endocardial catheter mapping (MEAM) is another valuable technique for evaluation of size of the heart cavities and studies have compared these results with echocardiographic measurements.^13^

The aim of our study was to assess left atrial size in patients with end-stage renal disease and determine the correlation between dimensions taken from different views using transthoracic echocardiography.

## Methods

The study population consisted of all patients on regular haemodialysis at the University Clinical Cente of Kosova in the first six months of 2007. We analysed data from transthoracic echocardiographic examinations performed with Doppler.

This was a cross-sectional study including all patients (except those specifically excluded) who underwent haemodialysis on a regular basis at the time of the study. Patients were divided randomly into two groups. Echocardiography was performed in one group prior to haemodialysis and in the other group after haemodialysis. The examination was done on the day the patients arrived for haemodialysis. The echocardiographic readings were performed on all patients by the same two operators.

A nurse took all blood pressure measurements using a mercury sphygmomanometer. The patients were asked to take their usual antihypertensive medications. Brachial blood pressure was measured twice in a sitting position after the patient had rested for more than 10 minutes. Blood pressure measurement was carried out in the contralateral arteriovenous fistula arm. Phase I and V of the Korotkoff sounds were taken as systolic and diastolic blood pressure, respectively, and averaged for two measurements. Classification of blood pressure was done according to the seventh report of the Joint National Committee on Prevention, Detection, Evaluation and Treatment of High Blood Pressure.^14^

Exclusions from this study were patients undergoing acute haemodialysis and those who presented with congenital or acquired valve disease. Patients with evidence of ischaemic heart disease as detected by changes in the ECG prior to enrolment in the study were also excluded.

Transthoracic echocardiography with Doppler was carried out in the long-axis view, short-axis view, apical four-chamber view, and apical two-chamber view. Apart from these standard measurements, the left atrium was examined from the anteroposterior long axis, orthogonal apical four-chamber, orthogonal apical two-chamber, and orthogonal short-axis views. The area was calculated for each orthogonal dimension separately and the total was averaged. The left atrial volume (LAV) was calculated using the formulae:

$\frac{LAV=0.85\;\times A1\;\times A2}{L}$

where A1 is left atrial area in the apical four-chamber view, A2 is left atrial area in the apical two-chamber view, and L is orthogonal (vertical) left atrial dimension in the apical fourchamber view.

$LAV=\left(D1\;\times D2\;\times D3\right)\;\times 0.523$

where D1 is the anteroposterior left atrial dimension in the longaxis view, D2 is the orthogonal (vertical) left atrial dimension in the apical four-chamber view, and D3 is the orthogonal (horizontal) left atrial dimension in the apical four-chamber view.

## Results

The total number of patients examined was 123 with a mean age of 56.39 (± 13.86) years and 56.1% were female. The most representative age group was 60 to 69 years (38.2%) (Table 1).

**Table 1:** Group Ages And Genders Of The Analysed Haemodialysis Patients

*Age group (years)*	*Female*		*Male*		*Total*	
	*n*	*%*	*n*	*%*	*n*	*%*
≤ 19	2	2.90	–	0.00	2	1.63
20–29	2	2.90	5	9.26	7	5.69
30–39	6	8.70	2	3.70	8	6.50
40–49	10	14.49	6	11.11	16	13.01
50–59	18	26.09	8	14.81	26	21.14
60–69	22	31.88	25	46.30	47	38.21
70+	9	13.04	8	14.81	17	13.82
*x*-bar	55.52		57.50		56.39	
SD	13.47		14.40		13.86	
Total: *n*	69	100.00	54	100.00	123	100.00
%	56.10	–	43.90	–	100.00	–

*t*-test: *p* > 0.05

The mean period of haemodialysis was 5.4 (± 3.4) years; 65% of the patients had been on dialysis for less than five years, and 29.3% for six to 10 years (Table 2). All patients had endstage renal disease and the underlying causes of chronic renal failure were: chronic glomerulonephritis (30.0%), hypertension (30.0%), diabetes (20.0%), tubulo-interstitial nephritis (10.0%), and unknown causes (10.0%). Most patients had normal sinus rhythm, but those with atrial fibrillation were not excluded. Dilated left atrium was found 32 patients (26.02%) with endstage renal disease and 62.5% of these were in the group who had had the disease for less than 10 years (Table 3).

**Table 2:** Time From The Start Of Haemodialysis

*Time from first haemodialysis (years)*	*Female*		*Male*		*Total*	
	*n*	*%*	*n*	*%*	*n*	*%*
0–5	42	60.9	38	70.4	80	65.0
6–10	24	34.8	12	22.2	36	29.3
> 10	3	4.3	4	7.4	7	5.7
Total	69	100.0	54	100.0	123	100.0
*x*-bar	5.3		5.5		5.4	
SD	3.2		3.7		3.4	

*t*-test: *p* > 0.05

**Table 3:** Dilated Left Atrium In Our Study Population

*End-stage renal disease (years)*						
	*< 10*		*> 10*		*Total*	
*Modalities*	*n*	*%*	*n*	*%*	*n*	*%*
Female	8	40.0	5	41.7	13	40.6
Male	12	60.0	7	58.3	19	59.4
Age group (years)					
20–29	0	0.0	1	8.3	1	3.1
30–39	1	5.0	0	0.0	1	3.1
40–49	2	10.0	1	8.3	3	9.4
50–59	2	10.0	3	25.0	5	15.6
60–69	12	60.0	5	41.7	17	53.1
70+	3	15.0	2	16.7	5	15.6
Total	20	100.0	12	100.0	32	100.0
	62.5	–	37.5	–	100.0	–

In our study there was a significant medium correlation (*r* = 0.60) between left atrial size and atrial volume using formula 1 (Table 4), and a significant high correlation (*r* = 0.89) between left atrial size and volume using the ellipsoid method (formula 2) (Table 5). Using both formulae, there was a significant medium correlation (r = 0.75) between left atrial volumes calculated (Table 6).

**Table 4:** Correlation Between Left Atrium Size And Its Volume

*Total*	*x: LA*	*y: LAVa*
sum	4522	3642.95
*n*	123	123
*xb*	36.76	29.62
SD	5.94	9.62
*r*	0.60	-
T	8.16	-
*a*	–5.8	-
*b*	1.0	-
y = a + bx	y = 1.0x − 5.8	

LA = left atrium, LAVa = left atrial volume using formula 1.

**Table 5:** Correlation Between Left Atrium Size And Its Volume

*Total*	*x: LA*	*y: LAVb*
sum	4522	3621.98
*n*	123	123
*xb*	36.76	29.45
SD	5.94	14.48
*r*	0.89	–
T	21.18	–
*a*	–50.0	–
*b*	2.2	–
y = a + bx	y = –50.0 + 2.2x	

LA = left atrium , LAVb = left atrial volume using formula 2.

**Table 6:** Correlation Between Calculated Volumes Of The Left Atrium

*Total*	*x: LA*	*y: LAVb*
sum	3642.95	3621.98
*n*	123	123
*xb*	29.62	29.45
SD	9.62	14.48
*r*	0.75	–
T	12.62	–
*a*	–4.15	–
*b*	1.13	–
y = a + bx		

LAVa = left atrial volume using formula 1, LAVb = left atrial volume using formula 2.

The mean value for left atrial size determined from the parasternal long-axis view was 36.76 ± 5.94 mm, and from the parasternal short-axis view it was 34.29 ± 5.87 mm (Table 7). The highest values for atrial size were obtained from the apical four-chamber view, with a mean of 42.96 ± 7.92 mm, and the apical two-chamber view with a mean of 39.18 ± 8.32 mm (Tables 8 and 9).

**Table 7:** Correlation Of Left Atrial Dimensions From Parasternal Transthoracic View

	*Female*		*Male*		*Total*	
	*y*	*x*	*y*	*x*	*y*	*x*
*Total*	*SA*	*LA*	*SA*	*LA*	*SA*	*LA*
sum	2289	2471	1929	2051	4218	4522
*n*	69	69	54	54	123	123
*xb*	33.17	35.81	35.72	37.98	34.29	36.76
SD	5.32	5.65	6.26	6.14	5.87	5.94
*r*	0.86	–	0.90	–	0.88	–
T	13.88	–	14.67	–	20.69	–
*a*	4.1	–	1.0	–	2.25	–
*b*	0.8	–	0.9	–	0.87	–
y = a + bx	y = 4.1 + 0.8x		y = 1.0 + 0.9x		y = 2.25 + 0.87x	

SA = short-axis view, LA = left atrium.

**Table 8:** Correlation Between Left Atrial Dimensions From Parasternal Long-Axis And Apical Four-Chamber Views

	*Female*		*Male*		*Total*	
	*y*	*x*	*y*	*x*	*y*	*x*
*Total*	*4C*	*LA*	*4C*	*LA*	*4C*	*LA*
sum	2896	2471	2388	2051	5284	4522
*n*	69	69	54	54	123	123
*xb*	41.97	35.81	44.22	37.98	42.96	36.76
SD	8.32	5.65	7.24	6.14	7.92	5.94
*r*	0.64	–	0.59	–	0.62	–
T	6.76	–	5.24	–	8.73	–
*a*	8.37	–	17.90	–	12.52	–
*b*	0.94	–	0.69	–	0.83	–
y = a + bx	y = 8.37 + 0.94x		y = 17.90 + 0.69x		y = 12.52 + 0.83x	

4C = four-chamber view, LA = left atrium.

**Table 9:** Correlation Between Left Atrial Dimensions From Parasternal Long-Axis And Apical Two-Chamber Views

	*Female*		*Male*		*Total*	
	*y*	*x*	*y*	*x*	*y*	*x*
*Total*	*2C*	*LA*	*2C*	*LA*	*2C*	*LA*
sum	2652.3	2471	2167	2051	4819.3	4522
*n*	69	69	54	54	123	123
*xb*	38.44	35.81	40.13	37.98	39.18	36.76
SD	8.92	5.65	7.46	6.14	8.32	5.94
*r*	0.60	–	0.65	–	0.62	–
T	6.21	–	6.12	–	8.74	–
*a*	4.28	–	10.27	–	7.18	–
*b*	0.95	–	0.79	–	0.87	–
y = a + bx	y = 4.28 + 0.95x		y = 10.27 + 0.79x		y = 7.18 + 0.87x	

2C = two-chamber view, LA = left atrium.

## Discussion

Anteroposterior measurements of the left atrial dimensions are generally used in everyday clinical practice and during study protocols, but since there is no standardised method of measurement, there is some uncertainty on the accuracy, within accepted norms, of calculations of left atrial volume.

Some studies have taken measurements of the left atrium during ventricular end-diastole. These include M-mode anteroposterior dimensions in the parasternal long-axis view, digitalised planimetry in the apical four-chamber view, and digitalised planimetry in the apical two-chamber view.^15^ Volumes were calculated for a spherical form in the first two methods and for a disc form in the last technique.

The correlation coefficient for calculations of left atrial volume using M-mode and two-dimensional approaches was *r* = 0.76. The mean difference between these two methods was 25 ± 33 ml (± 2 SD). Correlation between the values obtained with the four-chamber apical view and the two-dimensional method was *r* = 0.97. The mean difference between these two methods was 5.0 ± 12 ml (± 2 SD), which is very acceptable. The authors of this study concluded that measurements of left atrial size using M-mode echocardiography were not as representative as those determined by two-dimensional methods. M-mode measurements should therefore be used with caution or not at all.^15^

Gradin *et al.* in their study found a significant correlation between measurements of the left atrium from the parasternal and apical views, but values from the apical view were higher. They found large variations in the dimensions of the left atrium between different subjects and no correlation between these measurements and body and chest size, or diagnosis. They recommended that measurements be taken from both parasternal and apical positions, and that the upper limit of left atrial size from the apical view should be 45 mm.^12^

In our study, there was a high correlation between the different measurements of the left atrium for the total patient population and the correlation was also gender related. A moderate correlation resulted from measurements in all patients from the parasternal long-axis (*r* = 0.88) and apical four-chamber views from one side (*r* = 0.62), and the two-chamber view from the other side (*r* = 0.62).

Studies have compared different methods and approaches to measuring the left atrium using two- and three-dimensional echocardiography,^5^ echocardiography with digital views,^16^ and magnetic resonance,^6^ in order to find an accurate approach that can be used in routine daily practice.^17^ Some studies recommend M-mode echocardiography for patients with atrial fibrillation that is refractory to medication.^13^

## Conclusions

A dilated left atrium was found in almost one-third of the study population, with a larger number of these cases presenting with up to 10 years of disease duration. There was a high correlation between the size of the left atrium and its volume, calculated by area and the ellipsoid formula, respectively, with gender.

A high correlation was found between left atrial dimensions resulting from the two parasternal views. A moderate correlation was seen between left atrial dimensions from the parasternal long-axis view and the apical four- and two-chamber views. The highest values for left atrial size resulted from the apical fourchamber view. Evaluation of left atrial size should therefore be based on several measurements taken from different echocardiographic views.

The study assessed atrial dimensions in patients on haemodialysis using additional, non-conventional methods, and determined the relationship between these dimensions in order to better evaluate atrial size. We correlated these measurements of the left atrium, but did not include the eventual changes in atrial size caused by factors such as ongoing disease. An analysis of the latter may follow in another article.
